# Balanced performance measurement in research hospitals: the participative case study of a haematology department

**DOI:** 10.1186/s12913-017-2479-6

**Published:** 2017-08-03

**Authors:** Simona Catuogno, Claudia Arena, Sara Saggese, Fabrizia Sarto

**Affiliations:** 1 Department of Economics, Management, Institutions, University of Naples “Federico II”, Via Cinthia, Monte S. Angelo, 80126 Naples, Italy; 2 Department of Clinical and Experimental Medicine, Magna Græcia University of Catanzaro, Viale Europa, Catanzaro, 88100 Italy

**Keywords:** Performance measurement system, Research hospital, Case study, Balanced scorecard

## Abstract

**Background:**

The paper aims to review, design and implement a multidimensional performance measurement system for a public research hospital in order to address the complexity of its multifaceted stakeholder requirements and its double institutional aim of care and research.

**Method:**

The methodology relies on a participative case study performed by external researchers in close collaboration with the staff of an Italian research hospital.

**Results:**

The paper develops and applies a customized version of balanced scorecard based on a new set of performance measures. Our findings suggest that it can be considered an effective framework for measuring the research hospital performance, thanks to a combination of generalizable and context-specific factors.

**Conclusions:**

By showing how the balanced scorecard framework can be customized to research hospitals, the paper is especially of interest for complex healthcare organizations that are implementing management accounting practices. The paper contributes to the body of literature on the application of the balanced scorecard in healthcare through an examination of the challenges in designing and implementing this multidimensional performance tool. This is one of the first papers that show how the balanced scorecard model can be adapted to fit the specific requirements of public research hospitals.

## Background

In most developed countries, the healthcare sector is one of the fastest growing settings of the economy. Healthcare systems are especially complex as they are characterized by heterogeneous set of entities, activities and processes due to the involvement of citizens, clinicians, government, patients and professionals. Healthcare organizations can be described as based on three domains related to policy, administrative management, and professional services [1, 2]. Each domain operates on different and contrasting principles, success measures, structural arrangements, and work modes, and can be seen as conflicting with each other [3]. This complexity is further enhanced by the wide range of clinicians (doctors with different specialty and nurses) carrying to the system a different set of needs, priorities and evaluation criteria. In addition, these organizations also face important challenges due to the increased patient empowerment [4].

In healthcare, the above-mentioned presence of numerous stakeholders, on the one side intensifies the demand of information for supporting the appropriateness of their decisions. On the other side, it raises the need of measuring multiple performance dimensions to satisfy the interests of different stakeholders [5, 6]. In this sense, the design of an effective performance measurement system that fosters information sharing and accountability, and that includes the selection of multiple measures for analysing results, is central. Indeed, performance measurement is especially useful for benchmarking, rewarding quality and efficiency [7] and helping professionals in evaluating clinical practice [8].

Nevertheless, many organizations fail to address effectively these issues. Recent literature strongly emphasizes the importance of choosing indicators that are meaningful, strategic and evidence-based. Moreover, scholars highlight the need to complement traditional financial indicators with non-financial performance measures in order to satisfy the manifold accountability requirements of stakeholders [2, 9]. Indeed, much of the criticism of focusing only on financial performance measures stems from the idea that they may diminish prospects for overall improvement, due to the complex and multifaceted purposes of healthcare organizations [10, 11]. For instance, while patients may only feel competent to appreciate the clinical process with which they are handled (e.g. waiting times, length of stay), clinical staff could be more concerned with measures of medical outcomes (e.g. re-infection rates) and administrative staff with measures of outputs (e.g. number of patients handled, bed occupancy, financial return achieved). To achieve an acceptable performance level in every area, literature claims that it is necessary to effectively measure each of them [12].

To deal with these criticisms, authors have proposed different performance measurement models for healthcare organizations, suggesting that an ideal framework should include a balanced set of financial and non-financial interrelated dimensions [13–15]. Scholars highlight that, among the most widely used models (e.g. the performance pyramid, balanced performance measurement matrix, results and determinants framework) [16, 17], the balanced scorecard (hereafter BSC) is the only one that makes explicit links between different performance dimensions for the evaluation of a complex system, such as healthcare organizations [18]. Indeed, it satisfies the accountability request of the various stakeholders characterized by individual preferences, purposes and values. Moreover, it provides the identification of a limited number of key performance indicators (hereafter KPI), which in turn supports a clear strategic focusing in such a complex operating environment [19, 20].

Empirical research has provided case study evidence on the BSC’s adoption in healthcare [21]. In particular, they have focused on hospitals, university departments, psychiatric centres and national healthcare organizations [22]. This line of research points out that the unique characteristics of healthcare organizations may mitigate the benefits of the traditional framework [23]. Indeed, these entities distinguish themselves for mission (profit vs not for profit, teaching vs research), size, clinical specialty (acute vs not acute care; mono-specialist vs general) and services [3, 24, 25].

Moving from these considerations, a number of studies have found useful to make modifications to the Kaplan and Norton’s original formulation [25, 26]. In this regard, two research streams have emerged discussing how many and which perspectives should be included in the framework [27–29]. The former comprises studies devoted to add or modify perspectives [23, 30], and points out that the quality of care and its outcomes should be included in the basic structure of the BSC. The latter provides case study evidence that completely revises the original framework both in terms of number and types of perspectives [31–33].

However, while there is a broad range of literature on performance evaluation systems in public general hospitals [34], there are few papers exploring the application of performance measurement system (hereafter PMS) within public research ones. Our paper aims to fill this gap by developing a balanced performance measurement tool within the haematology department of an Italian research hospital (hereafter RH). In particular, the paper presents a participative case study [35, 36] performed by external researchers in close collaboration with the staff at the division. Indeed, the research team has been involved in a process of review, design and implementation of a multidimensional performance measurement system. The use of a participatory approach is especially useful in complex organizations as RHs, due to their double institutional aim of care and research. In fact, the achievement of this twofold scientific mission prevents the implementation of PMS especially in the light of the recent challenges of public healthcare sector (i.e. evolution of patient demand, technological sophistication, improvement of service outcomes and financial pressures on national budget) [37, 38].

The outline of the paper is the following: section 2 reports the research design; section 3 presents the development and the implementation of the PMS; section 4 concludes by providing theoretical and practical implications as well as directions for future research.

## Method

### Study design

We use a qualitative methodology based on a participative case study [35, 36, 39–41] since it is particularly useful for exploring complex organizational phenomena [42]. Differently from the action research which mainly aims to improves practices [43], participative case studies contribute to existing knowledge by deepening or widening the current understanding of the phenomenon under investigation, especially in early stages of research where prior evidence is lacking and existing theory seems inadequate [39, 40, 44, 45].

The case study is performed in close collaboration with the staff of the haematology department of an Italian RH by following the participatory approach. It helps organizations to develop and implement management practices, such as PMSs, when there are problems related to their implementation [36].

The authors were actively involved in the project carried out as part of a departmental project in management accounting. In particular, the project was developed at the haematology department of a public RH in the south of Italy. The choice to focus on a RH is connected to its involvement in a continuous performance improvement process. As part of these efforts, in 2014, the clinical director committed to the authors the development of a PMS to appreciate the trend of departmental performance.

The research design was based on a three steps procedure.

In the first step, we evaluated the existing PMS to identify, describe, and analyse the measures used by the RH. To this aim, we conducted semi-structured interviews and collected data from the information systems, technical reports, and internal documents of the RH [46–48]. A total of 14 interviews were carried out (i.e. eight to the clinical directors and six to the clinicians). They were guided by surveys and were conducted according to the principles of the performance measurement literature [49–51]. Moreover, they were carried out based on an interview schedule that was tested on a subsample of 4 interviewees belonging to the two categories analysed. The interviews were digitally recorded and then typed. Where necessary, we set up a second shorter interview to clear up information arisen in other interviews. At the end of this procedure, following prior literature, two co-authors (one of whom did not undertake the interviews) cross-coded the collected information in an attempt to address any inherent bias and subjectivity in the coding and analysis [52].

In the second step, a multidimensional PMS was developed. In particular, we decided to customize the BSC model that is considered the most appropriate tool for capturing the multifaceted performance of complex organizations [53]. Therefore, we analysed the strategic plans of the RH and of the department in order to identify their strategic objectives. Moreover, relying on previous BSC literature efforts in healthcare [54–57], we identified the most appropriate performance indicators for our setting according to their scientific soundness (i.e. reliability and validity), relevance (i.e. usefulness to managers and providers) and feasibility (i.e. ability to describe frequent activities or events so as to ensure meaningful comparisons) [58]. To finalize the identification of indicators, we restricted the performance metrics according to the information collected through the interviews and the meetings with the same interviewees involved in the first step [58, 59]. Indeed, we interactively identified and adjusted the indicators according to the availability of information regarding strategy, processes and patients. The selected measures were listed in a comprehensive handbook and validated by the interviewees. Furthermore, they were stored in a continuously updated electronic spreadsheet in order to make the process replicable for other researchers. The interviews were conducted according the same collection tactic described above.

In the third and final step of our methodology, we tested the BSC. To this aim, we analyzed the critical events occurred over the last decade at the RH department. At the end of this procedure, we identified the settlement of the partnership with a charity institution, established in 2009, as the most significant one. Thereby, we decided to compare the performance changes occurred over the 2 years before (2007–2008) the settlement of the partnership and the last two available years (2014–2015). Data for computing indicators were drawn from multiple sources: hospital discharge database, charity and departmental reports as well as questionnaires. These last tools are especially appropriate to test the stakeholders’ satisfaction since their dynamics are less straightforward in healthcare than in other settings [60, 61].

### The study setting: The research hospital

The RH under scrutiny is an Italian mono-specialist hospital that provides oncological cares.

RHs are ‘hospitals with a scientific purpose’ that combine clinical and research activities, mainly funded by the Italian Ministry of Health. On the one side, they provide an outstanding level of care [62]. On the other side, they directly contribute to the research priorities of the NHS as they conduct research that is likely to provide better care for patients [63]. The co-presence of the double institutional aim, and the duty of accountability as counterpart of additional financial resources provided by the Ministry of Health, make RHs as complex organizations [38, 64]. In addition, they are of smaller size in comparison to teaching and general hospitals, as they are organizations that focus intensively on their research activity and offer limited but specialized cares to patients [65]. As a result, their case-mix is usually higher than the general and teaching ones [38, 66]. Moreover, as additional feature of complexity, RHs frequently establish collaborations with private organizations for fund raising purposes as well as for the outsourcing of specific clinical activities (such as home care services).

As previously stated, this paper focuses on the haematology department of a southern Italian RH that represents one of the centres of excellence in this setting in providing cares for haematology-oncological disorders. Indeed, it presents 12 beds for ordinary hospitalization, 6 beds for transplants, a day-hospital facility, a specialist laboratory and an ambulatory for both the follow-up of discharged patients and those under experimental treatments.

One of the most relevant features of the department is that, since 2009, its activities are supported by the partnership with a charity institution aiming to provide home care assistance. In particular, under the Framework Law on Volunteering (clause 7, law n. 266/1991), the department signed a convention with an Italian charity, aimed at raising funds to finance the delivering of home care services for the patients treated at the department through a multi-professional team (i.e. doctors, nurses, psychologists). The home care program includes activities such as blood transfusion, side effect treatment and home chemotherapy, all delivered by the clinical staff employed at the hospital. Aside the home care, the charity also provides a range of services at the department to support the patients and their families (i.e. infrastructure empowerment, scientific equipment, medicines, and on demand TV packages). In addition, the charity funds a psychological service to both patients and their families in order to improve their quality of life. The main channels of financing are donations (by private individuals, companies, bank foundations and governmental agencies), charity gala dinner and jumble sales.

## Results

### The analysis of the existing PMS of the research hospital

The interviewees and the data collection highlighted that the RH relied on a simplified PMS based on the following tools: strategic plan, annual report, budget plan, performance plan based on past and expected performance data.

The annual report complied with the civil code and the Italian and International GAAP, as well as the decree 16.02.2001 of the Italian Ministry of Health. It presented the past economic and financial performance data as well as a master budget designed to present a complete picture of the economic and financial activity of the RH.

The performance plan was structured in three main sections. The first presented general information on the identity of the hospital in terms of activity and institutional mission. The second described the normative settings in terms of legislation, demographic and economic framework. The third defined the strategic and operating targets and designed the actions and the processes aimed at improving the performance management cycle.

The PMS was based on two main categories of indicators. The former was related to the clinical activity (e.g. the total number of beds available at the department, number of total admissions, average length of stay), while the latter focused on the research activity (e.g. total number of publication, number of research project).

During the interviews process, a general dissatisfaction emerged with regard to the existing PMS. Indeed, the interviews emphasized that it was unable to capture both the non-financial performance and the effects of the partnership settled by the department.

### The development of the BSC for research hospitals

In order to overcome the limitations emerged with the existing PMS, researchers developed a customized model of the BSC. To do this, we followed the standard practices of literature on most updated BSC approach, which recommend linking a balanced set of KPI to the strategy [67].

As already acknowledged by the literature, most interviewees to the clinicians and the director confirmed that the classical framework was inappropriate for the department and some changes were needed. In fact, in the traditional formulation of the BSC the economic and financial dimension is included at the top of the hierarchy. While this perspective is useful to evaluate the hospital ability to effectively fulfil its institutional mandate, a primary focus on it may actually hinder organizational growth and success [10]. Indeed, literature suggests that positioning financial objectives at the top of the BSC, which is typical of the classic conceptualization in private organizations, appears inconsistent with the aims of public hospitals [16, 30, 31]. Differently, proponents of this model suggest that the BSC can be successfully applied in healthcare by placing customer or constituent perspectives at the top of the hierarchy [68]. Moreover, scholars emphasize that the selection of BSC perspectives should also account for the institutional mission of the hospital [16, 23]. For instance, when applying the BSC to the teaching hospitals, some authors integrated the BSC with the teaching perspective [69]. With this in mind, we revised the Kaplan and Norton’s BSC and decided to place the stakeholder perspective at the top of the model. In addition, we introduced the care and research processes as specific dimensions able to catch the twofold institutional aims of the RH [70].

**Fig. 1:**
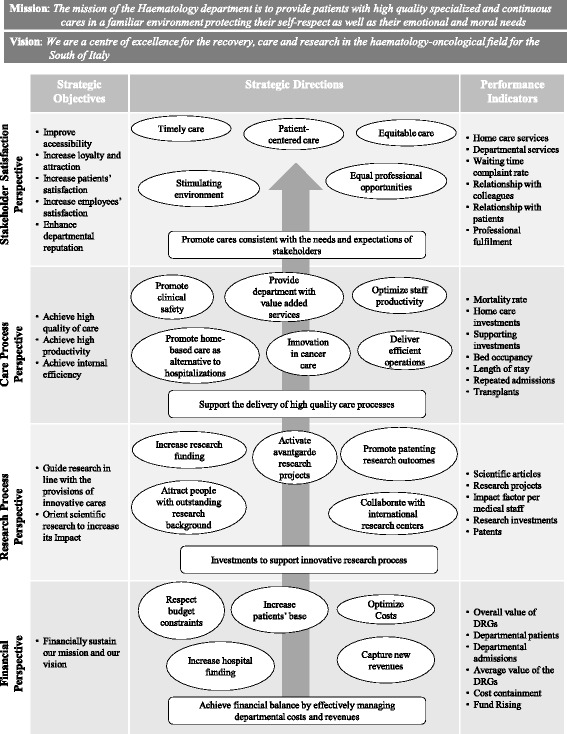
From the strategic map to the BSC

Starting from the departmental 5 years strategic plan (included in the RH strategic plan) and the departmental budget, we identified the most relevant strategic objectives and the related strategic directions and classified them in the four perspectives of our BSC [67]. Based on the strategic objectives and directions, for each perspective we developed a set of performance indicators according to the criteria illustrated in the methodological section, validated by the interviewees [58] (see Fig. 1).

This procedure led to our BSC that matches the perspectives with the key performance activities (hereafter KPAs) and indicators (KPIs). The novelty of the developed tool consists of complementing the traditional input/output measures (e.g. bed occupancy, length of stay and numbers of discharges and admissions) [71–73] by selecting the indicators that can also capture the multifaceted effects of the partnership on the departmental performance (e.g. the satisfaction of employees, both in terms of doctors and nurses; the departmental fund rising on the total available resources).

With regard to the *stakeholder satisfaction* perspective, researchers decided to rely on two KPAs. The first one is the patient satisfaction. Within this KPA, we identified the following KPIs: satisfaction degree about (a) the home care services and (b) the departmental services; (c) waiting time; and (d) complaint rate. As emerged by the discussion with the clinicians, these KPIs reflect the two fundamental requirements of stakeholders, i.e. the timely provision of proper cares and the support for the follow-up caring services. As for the second KPA, we identified the satisfaction of employees (doctors and nurses). Within this KPA, we selected the following KPIs: (e) satisfaction degree about the relationship with colleagues; (f) relationship with patients and; (g) professional fulfilment. The satisfaction degrees were based on Likert scale (1–4) and data were collected through a survey.

Regarding the perspective of the *care processes*, researchers decided to select one KPA that covers the quality, productivity and internal efficiency. As for the KPIs, it is worth noting that the discussion with clinicians highlighted that the mortality remains the predominant traditional outcome measure. Nevertheless, it presents the disadvantage of reflecting a rare and end-stage event. Hence, we decided to complement (h) the mortality rate with other six KPIs, i.e. (i) investments in home care services and (j) supporting services (medicines, psychological counselling, furniture, secretary services, TV colour, on demand TV packages); (k) bed occupancy; (l) length of stay; (m) repeated admissions; and (n) transplants.

As for the perspective of the *research processes,* we considered two KPAs. The former covers the outcomes of scientific research. The related KPIs refer to (o) scientific articles published in national and international journals, (p) existing financed research projects, (q) impact factor (IF). Differently, the latter KPA catches the innovative process of the department both in terms of (r) research investments and (s) patents, as the related KPIs.

Finally, as for the *economic and financial* perspective, researchers focused on the trend of revenues and costs. As for the former KPA, they identified four KPIs: (t) overall value of diagnosis related group tariffs (DRGs as represented by the reimbursement that NHS pays to the department for the diagnosis group); (u) patients treated at the department; (v) admissions at the department; and (w) average value of the DRGs tariffs. As emerged by the validation process, with regard to the latter KPA, researchers decided to focus on (x) the cost containment as the KPI catching the average cost of the services delivered by the department. Differently, to appreciate the ability of the partner to deliver financial resources to the RH, we measured (y) the departmental fund rising.

Table 1 shows, for each KPA, the indicators and provides details of their measurement.

**Table 1:** Structure of BSC and source of data

Perspective	*KPA*		KPI	*Source of Data*	Description
Stakeholder satisfaction	*Patient*	a.	Home care services	*Questionnaire to home care patients*	Satisfaction degree by Likert scale (1–4)
		b.	Departmental services	*Questionnaire to home care patients*	Satisfaction degree by Likert scale (1–4)
		c.	Waiting time	*Departmental Report*	Average waiting time for treatment
		d.	Complaint rate	*Departmental Report*	Number of complaints per admissions
	*Employees*	e.	Relationship with colleagues	*Questionnaire to employees*	Satisfaction degree by Likert scale (1–4)
		f.	Relationship with patients	*Questionnaire to employees*	Satisfaction degree by Likert scale (1–4)
		g.	Professional fulfilment	*Questionnaire to employees*	Satisfaction degree by Likert scale (1–4)
Care processes	*Quality, productivity and internal efficiency*	h.	Mortality rate	*Hospital Discharge Database*	Number of death at the department/Number of patients
		i.	Home care investments	*Charity Report*	Amount of investments in home care services (€)
		j.	Supporting investments	*Charity Report*	Amount of investments in supporting services (€)
		k.	Bed occupancy	*Hospital Discharge Database*	(Number of admissions^b^ Average length of stay)/maximum productive capacity^a^
		l.	Length of stay	*Hospital Discharge Database*	Average length of stay
		m.	Repeated admissions	*Hospital Discharge Database*	Number of repeated admissions
		n.	Transplants	*Hospital Discharge Database*	Number of Transplants
Research process	*Scientific research*	o.	Scientific articles	*Departmental Report*	Average number of scientific articles in national and international journals per researcher per year
		p.	Research projects	*Departmental Report*	Number of existing financed research projects
		q.	IF per medical staff	*Departmental Report*	Average impact factor per medical staff
	*Innovative process*	r.	Research investments	*Departmental Report*	Amount of investments in research (€)/ Research Staff
		s.	Patents	*Departmental Report*	Number of patents per medical staff
Economic and financial	*Revenues*	t.	Overall value of DRGs	*Hospital Discharge Database*	Reimbursement that NHS pays to the department for the diagnostic group
		u.	Departmental patients	*Hospital Discharge Database*	Number of patients treated at the Department
		v.	Departmental admissions	*Hospital Discharge Database*	Number of admissions at the Department
		w.	Average value of the DRGs	*Hospital Discharge Database*	Average value of the DRGs tariffs/number of admissions at the Department
	*Costs*	x.	Cost containment	*Departmental Report*	Total departmental operating costs/Total DRGs
		y.	Fund Rising	*Charity Report*	Total departmental fund rising/Total available resources

^a^n. beds

^b^365

### The application of the BSC to the research hospital

**Fig. 2:**
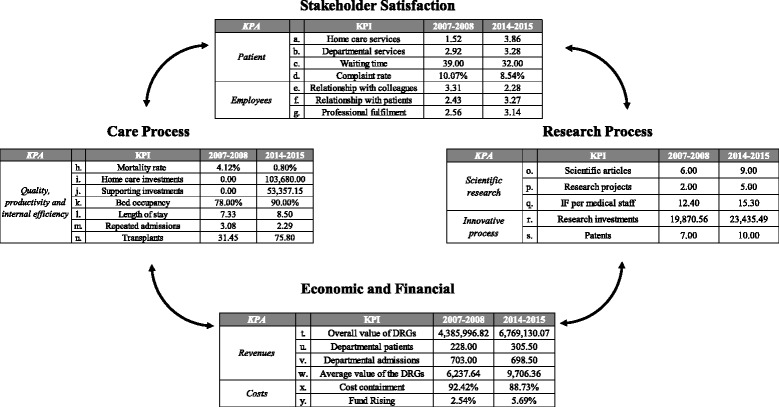
Application of BSC to the RH

The feasibility and usefulness of the BSC for the department was tested by applying the model in order to compare the performance indicators before and after the settlement of the partnership. Indeed, as shown in Fig. 2, the BSC pointed out the performance changes occurred over the 2 years before the partnership (2007–2008) and the last two available years (2014–2015).

**Fig. 3:**
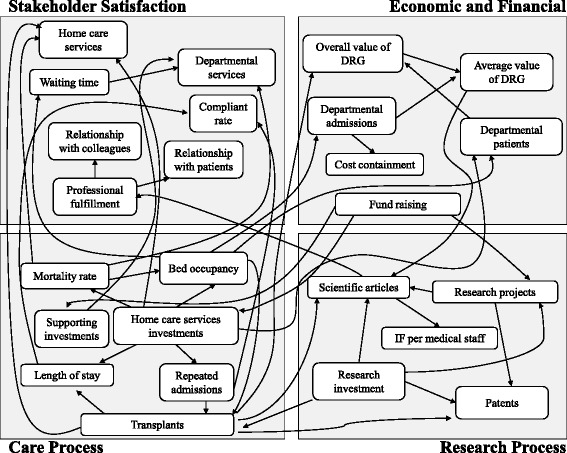
Map of relationships among KPIs

In order to emphasize the relationships among the perspectives and the related KPIs of our BSC, researchers also developed a map. Figure 3 shows that all the four performance perspectives of our model are closely interconnected. In particular, the arrows highlight the cause-and-effect relationships among the indicators. As shown by the figure, these relationships can be direct and/or indirect involving an intermediate KPI. For example, the home care service investments directly improve the bed occupancy and indirectly reduce the waiting time.

A closer look to the figure shows the most relevant relationships among KPIs within and across perspectives. In particular, we observe that the delivery of cares at the patients’ home (i.e. home care services investments) reduces the average number of repeated admissions at the department and increases the availability of beds for caring news patients (i.e. bed occupancy). As a result, the number of transplants increases in turn. It is worth noting that the treatment of these more complex diseases calls for outstanding research able to deal with the need of sophisticated cares. Thereby, it affects the research process performance as shown by the growing number of scientific articles in national and international journals authored by the clinicians staffed at the department.

The changes occurred in the care and research perspectives reflect into the KPIs belonging to the economic and financial as well as the stakeholder satisfaction dimensions. As for the former, we can note an improvement of the overall and the average values of DRG tariffs due to the recombination of admissions characterized by different DRGs. In fact, on the one hand the treatment of side effects of toxicity diseases and terminal illnesses (associated with lower DRG tariffs) is provided at home. On the other hand, it is replaced with transplants (associated with higher DRG tariffs). As for the latter, the limited discomfort of repeated admissions improves the patient satisfaction for the home care program. At the same time, the investments in supporting activities also increase the patient satisfaction for the departmental services. In fact, the empowered facilities contribute to the improvement of the overall comfort at the department. In addition, the patients’ stay benefits from the absence of terminally ills (i.e. mortality rate) in the departmental rooms.

Finally, concerning the employee satisfaction, the opportunity to spend more time on outstanding research (i.e. scientific articles) fosters the ability of the clinicians staffed at the department to provide more sophisticated cares (e.g. transplants) and increases their job satisfaction of (i.e. professional fulfilment). Thereby, these changes positively reflect into better relationship with colleagues.

## Discussions and conclusions

Among healthcare providers, RHs are complex organizations aiming to combine hospital care and research activities [38, 62, 64]. In this paper, we present the results of the design and implementation of a multidimensional PMS in a public RH based on a case study conducted through a participatory approach. The review of the existing PMS and the characteristics of the RH suggested the development of a customized version of BSC based on a new set of performance measures. In line with prior literature [74], we applied the proposed BSC by using historical data in order to validate the model. The departmental director and clinicians agreed upon the usefulness of the BSC as it allowed to appreciate the departmental performance dynamics over the period under scrutiny.

In this sense, the proposed BSC can be considered a useful framework for the measurement of the RH performance, thanks to a combination of generalizable and context-specific factors [75]. The former relate to the overall design and implementation process of the BSC. These factors include the stimulation of a participative climate among hospital directors and clinicians with periodical meetings; the development of a comprehensive handbook with the list of the performance indicators that reflect the critical performance areas; the continuous information updating as input of performance indicators. The latter relate to the industry and organizational-specific factors of the entity. They concern the structure of the BSC model in terms of type and positioning of performance dimensions. In this sense, the performance indicators should be clustered in different perspectives, reflecting the healthcare industry and the RH peculiarities. More specifically, our research emphasizes the importance of positioning the stakeholder perspective at the top of the BSC as well as introducing specific performance dimensions and distinctive KPAs and KPIs able to capture the care and research processes.

In this regard, our paper differentiates from previous research on BSC that has been developed in other settings. In particular, in various healthcare contexts, especially in North America, there have been several attempts to adapt and implement the BSC framework. In Ontario, for example, a number of hospitals have collaborated with university-based research teams to develop a BSC able to catch their performance. Overall, the identified indicators have been mainly aggregated in four areas: (i) clinical, utilization and outcomes, (ii) patient satisfaction, (iii) system integration and change, (iv) financial performance and conditions [58]. Over the past years, other organizations in Ontario (e.g. Cancer Care Ontario [76], Ontario Hospital Association [77] and University Health Network [78]) have slightly modified the original BSC framework of Kaplan and Norton to measure public health performance in four performance quadrants: (i) financial, (ii) customer preferences, (iii) business processes, (iv) learning and growth. Starting from these efforts, the Institute for Clinical Evaluative Science (ICES) released a report that introduced a public health specific BSC framework for performance measurement based on the following quadrants: (i) health determinants and status; (ii) community engagement; (iii) resources and services; (iv) integration and responsiveness [79]. The above mentioned model gives more room to the importance of catching hospital performance in terms of implications of initiatives for the community that uses local public health services. Opposite conclusions can be drawn for Italy. In this setting, due to the lack of governmental guidelines, the duty of accountability is mainly towards the stakeholders directly involved in the hospital processes (i.e. patients and employees), thus neglecting the interests of the community as a whole.

Based on these premises, the paper provides theoretical and practical contributions.

With regard to the theoretical one, it extends prior literature on multidimensional PMSs in healthcare. Moreover, it fills a gap in the empirical research by tailoring a BSC model to an underexplored setting, i.e. the public RH. The study has also additional value for practitioners in that it contributes to the ongoing debate regarding the performance measurement in healthcare complex settings. Indeed, thanks to the participatory approach, the paper highlights and overcomes the practical difficulties in implementing multidimensional PMSs in RH [50]. In addition, our map points out the virtuous circle among factors that play a crucial role for the RH’s outcomes and that should be closely monitored to improve its performance. Thereby, further research efforts should be devoted to improve the BSC model by broadening the stakeholder perspective to make the hospital accountable for its social responsibility towards the community.

Moreover, our BSC represents a useful starting point for policy makers to develop the existing performance evaluation tools of NHS at local level [65]. Finally, our paper broadens knowledge and offers evidence of a public hospital involved in a partnership. In this sense, on the one side, it sheds light on the advantages of settling collaboration with a private entity in terms of the RH’s performance. On the other side, the study underlines the usefulness of the BSC in catching the multidimensional performance implications of the partnership. Finally, moving from the experiences of North American countries, the research suggests that policy makers should provide hospitals with governmental guidelines able to support the design and the adoption of the BSC.

## Abbreviations

BSC: Balanced scorecard
DRG: Diagnosis related group
IF: Impact factor
KPA: Key performance activities
KPI: Key performance indicators
NHS: National Health Service
PMS: Performance measurement system
RH: Research hospital
